# Development of Loop-Mediated Isothermal Amplification Rapid Diagnostic Assays for the Detection of *Klebsiella pneumoniae* and Carbapenemase Genes in Clinical Samples

**DOI:** 10.3389/fmolb.2021.794961

**Published:** 2022-02-09

**Authors:** Aurore C. Poirier, Dai Kuang, Bianca S. Siedler, Khushboo Borah, Jai W. Mehat, Jialin Liu, Cui Tai, Xiaoli Wang, Arnoud H. M. van Vliet, Wei Ma, David R. Jenkins, John Clark, Roberto M. La Ragione, Jieming Qu, Johnjoe McFadden

**Affiliations:** ^1^ Department of Pathology and Infectious Diseases, Faculty of Health and Medical Sciences, School of Veterinary Medicine, University of Surrey, Guildford, United Kingdom; ^2^ Department of Pulmonary and Critical Care Medicine, Ruijin Hospital, School of Medicine, Institute of Respiratory Diseases, Shanghai Jiao Tong University, Shanghai, China; ^3^ Faculty of Health and Medical Sciences, School of Biosciences and Medicine, University of Surrey, Guildford, United Kingdom; ^4^ Centre for Microbial Genomics and Animal Microbiome Research, Department of Pathology and Infectious Diseases, Faculty of Health and Medical Sciences, School of Veterinary Medicine, University of Surrey, Guildford, United Kingdom; ^5^ Department of Critical Care Medicine, Ruijin Hospital, School of Medicine, Shanghai Jiao Tong University, Shanghai, China; ^6^ State Key Laboratory of Microbial Metabolism, School of Life Sciences and Biotechnology, Shanghai Jiao Tong University, Shanghai, China; ^7^ Department of Medical Microbiology, University Hospitals of Leicester NHS Trust, Leicester, United Kingdom; ^8^ Department of Medical Microbiology, Epsom and St Helier University Hospitals NHS Trust, Carshalton, United Kingdom

**Keywords:** rapid diagnostics, antimicrobial resistance (AMR), *Klebsiella pneumoniae (K. pneumoniae*), carbapenemases genes, LAMP (loop mediated isothermal amplification)

## Abstract

*Klebsiella pneumoniae* is an important pathogenic bacterium commonly associated with human healthcare and community-acquired infections. In recent years, *K. pneumoniae* has become a significant threat to global public and veterinary health, because of its high rates of antimicrobial resistance (AMR). Early diagnosis of *K. pneumoniae* infection and detection of any associated AMR would help to accelerate directed therapy and reduce the risk of the emergence of multidrug-resistant isolates. In this study, we identified three target genes (*yhaI*, *epsL*, and *xcpW*) common to *K. pneumoniae* isolates from both China and Europe and designed loop-mediated isothermal amplification (LAMP) assays for the detection of *K. pneumoniae* in clinical samples. We also designed LAMP assays for the detection of five AMR genes commonly associated with *K. pneumoniae*. The LAMP assays were validated on a total of 319 type reference strains and clinical isolates of diverse genetic backgrounds, in addition to 40 clinical human sputum samples, and were shown to be reliable, highly specific, and sensitive. For the *K. pneumoniae*–specific LAMP assay, the calculated sensitivity, specificity, and positive and negative predictive values (comparison with culture and matrix-assisted laser desorption/ionization–time of flight mass spectrometry) were all 100% on clinical isolates and, respectively, of 100%, 91%, and 90%, and 100% when tested on clinical sputum samples, while being significantly faster than the reference methods. For the *bla*_*KPC*_ and other carbapenemases’ LAMP assays, the concordance between the LAMP results and the references methods (susceptibility tests) was 100%, on both pure cultures (*n* = 125) and clinical samples (*n* = 18). In conclusion, we developed highly sensitive and specific LAMP assays for the clinical identification of *K. pneumoniae* and detection of carbapenem resistance.

## Introduction

*Klebsiella pneumoniae* is a Gram-negative bacterium belonging to the Enterobacteriaceae family within the *Enterobacterales* order (Adeolu et al., 2016). *K. pneumoniae* can be found in the environment, associated with plants, soil, and water, but can also colonize a wide range of animal and human hosts, resulting in clinical manifestations including liver abscess, respiratory tract, urinary tract, gut, skin, and systemic infections (Podschun and Ullmann, 1998; Li et al., 2014). Furthermore, *K. pneumoniae* is a common pathogen implicated in human healthcare-associated and community-acquired infections and is becoming a significant threat to global public and veterinary health, because of its high rates of antimicrobial resistance (AMR) (Wyres and Holt, 2016). The most common AMR *K. pneumoniae* lineages, associated with the occurrence of hospital outbreaks and deaths worldwide, are those producing the *K. pneumoniae* carbapenemase (KPC), conferring resistance to penicillins, most cephalosporins, and carbapenems (Nordmann et al., 2009; Palzkill, 2018). More than 100 distinct acquired AMR genes have been identified in *K. pneumoniae*, and historically, it is the organism where most AMR genes were first detected, before becoming widespread in other Gram-negative bacterial pathogens (Holt et al., 2015; Wyres and Holt, 2016). With the advent of high-throughput whole genome sequencing and core genome multilocus sequence typing in the last decade, several genomic studies investigated the population structure of *K. pneumoniae* and the evolution of AMR clones (Bialek-Davenet et al., 2014; Holt et al., 2015). These studies demonstrated the direct transfer of AMR plasmids between *K. pneumoniae* and other *Enterobacterales* in isolates recovered from hospital environments (Wyres and Holt, 2016), and the wide association of *K. pneumoniae* with carbapenemase-producing genes *bla*_KPC_, *bla*_OXA-48-like_, and *bla*_NDM-1_, and the extended-spectrum beta-lactamase (ESBL) gene *bla*_CTX-M-15_ (Munoz-Price et al., 2013; Pitout et al., 2015; Zowawi et al., 2015; Wyres and Holt, 2016). The dissemination of these carbapenemase-producing and ESBL genes and more recently plasmid-mediated colistin resistance gene *mcr-1* (Liu et al., 2016) is of particular clinical concern. Currently, the choices for carbapenemase detection are phenotypic tests that require bacterial culture, such as the modified Hodge test (Bartolini et al., 2014) and disc diffusion tests using meropenem, phenyl boronic acid, or ethylenediaminetetraacetic acid (Bartolini et al., 2014; Sood, 2014); matrix-assisted laser desorption/ionization time-of-flight mass spectrometry (MALDI-TOF MS) (Ghebremedhin et al., 2016; Yu et al., 2018); carba NP test (Nordmann et al., 2012; Segawa et al., 2017) or its derivatives (Tamma and Simner, 2018); and the carbapenem inhibition test (van der Zwaluw et al., 2015; Kuchibiro et al., 2018). Molecular methods such as polymerase chain reaction (PCR) (Solanki et al., 2013; Moloney et al., 2019) and loop-mediated isothermal amplification (LAMP) (Solanki et al., 2013; Cheng et al., 2014; Nakano et al., 2015; Srisrattakarn et al., 2017; Funashima et al., 2018; Feng et al., 2021) have also been described, for more rapid diagnostics. Early intervention facilitated by rapid diagnostics would help to accelerate directed therapy, thus slowing down the dissemination of AMR and reducing the risk of the emergence of multidrug-resistant strains that have limited treatment options (Brogan and Mossialos, 2016; O’Neill, 2016; Piddock, 2016).

LAMP is a single-step nucleic acid amplification technique that is rapid, sensitive, and cost-effective. This technique can amplify a few copies of DNA into billions of copies within 30 min (Notomi et al., 2000; Diribe et al., 2014) and has been used to detect a wide range of viral, bacterial, and parasitic pathogens (Wong Y.-P. et al., 2018; Li et al., 2019; Tian et al., 2019; Vergara et al., 2020). LAMP is less sensitive to PCR inhibitors in poorly or nonprocessed samples, such as blood, sputum, and urine, making it suitable for use as a point-of-care diagnostic (Notomi et al., 2000; Nagamine et al., 2002; Kaneko et al., 2005; Parida et al., 2008; Francois et al., 2011; Dhama et al., 2014; Gadkar et al., 2018; Moehling et al., 2021). LAMP is performed at a constant isothermal temperature using specifically designed primers and a strand-displacement DNA polymerase (Notomi et al., 2000), and the amplified products can be detected by fluorometry, turbidity, and colorimetry (Wong Y.-P. et al., 2018; Moehling et al., 2021). LAMP amplification can also be detected by the measurement of by-products using hydroxynaphthol blue dye, calcein, or malachite green (Liu et al., 2012; Cheng et al., 2014; Kim et al., 2016; Lucchi et al., 2016; Srisrattakarn et al., 2017).

In this study, we identified target genes common to *K. pneumoniae* isolates from both China and Europe and designed LAMP assays for the detection of *K. pneumoniae.* We also designed LAMP assays for the detection of the most important classes of AMR genes in *K. pneumoniae*. The LAMP assays were validated on a total 319 strains (5 *K. pneumoniae* type strains, 167 *K. pneumoniae* clinical isolates, 32 non–*K. pneumoniae* type strains, and 115 non–*K. pneumoniae* clinical isolates) from the United Kingdom and China in addition to 40 clinical sputum samples. All the designed assays were shown to be reliable, highly specific, and sensitive.

## Materials and Methods

### Ethics Approval Statement

Ethical approval, including the waiver of informed consent of the clinical strains and samples collected in Ruijin Hospital, was approved by the Ethics Committee of Ruijin Hospital, School of Medicine, Shanghai Jiao Tong University, under approval no. RJ2019NO1-3. The research conformed to the principles of the Helsinki Declaration. The study involved no more than minimal risk to subjects, and no personal information was obtained.

### Bacterial Isolates and Growth Conditions

In the United Kingdom, type strains of *K. pneumoniae*: NCTC 13809, NCTC 13438, and NCTC 13439 (Supplementary Table S1); *Escherichia coli*: NCTC 14321, NCTC 13441, and ATCC 25922; *Enterobacter cloacae*: NCTC 13380 and NCTC 14322; *Salmonella enterica*: ATCC 14028, NCTC 05776, NCTC 10705, NCTC 13346, and NCTC 10532; *Acinetobacter baumannii*: ATCC Ab19606; *Acinetobacter lwoffii*: NCTC 05866; *Staphylococcus aureus*: NCTC 12981; MRSA NCTC 12493, NCTC 8325; and MRSA BAA-1680/United States of America 300; *Yersinia enterocolitica*: NCTC 12982; and *Streptococcus pyogenes*: NCTC 12696 were purchased from Public Health England (PHE) culture collections (National Collection of Type Cultures [NCTC]) and American Type Culture Collection (ATCC) (Supplementary Table S2).

*K. pneumoniae* isolates (*n* = 47) were obtained from patient samples in University Hospitals of Leicester NHS Trust and Epsom and St. Helier University Hospitals, as well as from sink drain traps of a Lancashire NHS hospital (Supplementary Table S1). *K. pneumoniae* isolates were cultured aerobically at 37°C for 16 h on *Klebsiella* selective agar and then subcultured onto Luria–Bertani agar. *S. enterica* isolates (*n* = 5) from the Leicester hospital were cultured aerobically at 37°C on brilliant green agar for 16 h and then subcultured onto Luria–Bertani agar (Supplementary Table S2). *S. aureus*, *Enterobacter* spp., *Pseudomonas* spp. (*P. putida* KT2440 (Franklin et al., 1981), *P. aeruginosa* PAO1 (Jacobs et al., 2003)), *Klebsiella oxytoca* (*n* = 9, from sink drain traps of a Lancashire NHS hospital), and *Acinetobacter* spp. strains were cultured aerobically at 37°C for 16 h on Luria–Bertani agar before use in the LAMP development and validation (Supplementary Table S2).

In China, type strains of *K. pneumoniae* HS11286 and RJF999, *E. coli* DH5α, *S. enterica* ATCC14028, *A. baumannii* ATCC19606, *P. aeruginosa* PAO1, and *S. aureus N315* were obtained from State Key Laboratory of Microbial Metabolism, Shanghai Jiao Tong University. One hundred twenty *K. pneumoniae* clinical isolates and 110 non–*K. pneumoniae* clinical isolates (including 5 *Klebsiella aerogenes*, 5 *K. oxytoca,* 20 *E. coli*, 20 *S. enterica*, 20 *A. baumannii*, 20 *P. aeruginosa,* and 20 *S. aureus*) from China were collected and identified in Ruijin Hospital Affiliated to Shanghai Jiao Tong University School of Medicine by MALDI-TOF and MS, using the Vitek 2 automated system (bioMérieux, Inc., Marcy l’Etoile, France) in accordance with the manufacturer’s instructions, and cultured aerobically at 37°C for 16 h on Luria–Bertani agar before use (Supplementary Table S2).

### Identification of Gene Targets for the Detection of *K. pneumoniae*

A total of 8,638 *K. pneumoniae* genomes, from all over the world, were downloaded from online databases (FTP site, GenBank, NCBI) with their associated metadata, and 7,320 were selected based on assemblies metrics (Supplementary Table S3), using Quast version 4.5 (Gurevich et al., 2013). Contigs that comprised fewer than 200 nucleotides were excluded. Only genome assemblies with a total size between 4 and 6 Mbp, an N50 of >50 kb, and L50 <20 were included. To identify suitable discriminatory gene targets for *K. pneumoniae*, 100 (50 from Europe and 50 from Asia) of the *K. pneumoniae* genome assemblies were selected for the next stage of the study (Supplementary Table S4). The selected genomes assemblies were representative of the prevalent sequence types. In addition, 50 *K. oxytoca* genomes assemblies (Supplementary Table S5) were downloaded from online databases (FTP site, GenBank, NCBI). These 150 genomes assemblies were compared as detailed below. Genome assemblies were annotated using Prokka V.1.14.5 (Seemann, 2014), and annotated features were used for construction of a pangenome using Roary version 3.12.0 (Page et al., 2015) with a 95% identity cutoff.

The *K. pneumoniae* and *K*. *oxytoca* pan-genomes were used to determine genes specifically associated with *K. pneumoniae* using Scoary (Brynildsrud et al., 2016). Genes were determined to be associated with *K. pneumoniae* if they occurred at a frequency of 100% in specific *K. pneumoniae* and of 0% in *K. oxytoca*. *K. pneumoniae*–associated genes were incorporated into a custom ABRicate (https://github.com/tseemann/abricate) database (Supplementary List S6 with ABRicate database) and used to screen a large collection of 7,320 *K. pneumoniae* genomes derived from all over the world (Supplementary Table S3), using a BLAST cutoff of 80% identity and 80% coverage. An 80% identity cutoff was chosen for BLAST to allow identification of discriminatory target genes while also detecting gene homologs or orthologs with high sequence identity, as potential homologs could confound diagnostic gene target selection. Following the ABRicate BLAST, an NCBI BLAST, with default parameters and excluding *K. pneumoniae*, was performed to find orthologs in other bacteria with sequence similarity, for each identified target gene.

### Preparation of Target DNA

DNA from *K. pneumoniae* isolates was extracted using the Wizard^®^ Genomic Purification Kit (Promega, United Kingdom). DNA concentration was measured using a Nanoquant plate on a Tecan 10M Spark microplate reader (Tecan, United Kingdom). For routine testing of *K. pneumoniae* by LAMP, two to three colonies of a pure culture of *K. pneumoniae* isolates were boiled in 200 µl of nuclease-free water to produce a lysate. Samples were stored at −20°C until use. For determining the limit of DNA concentration of detection of the optimized LAMP assay, DNA was 10-fold serially diluted from 1 ng/μl to 10 fg/μl in nuclease-free water. The limit of bacterial copies per LAMP reaction was also determined, using a protocol previously described in other studies (Goto et al., 2010; Manajit et al., 2018). For these studies, a 16 h broth culture of *K. pneumoniae* HS11286 (OD600 nm at 0.5) was serially diluted 10-fold dilutions in 0.85% of normal saline. 100 µl of each dilution was plated on Luria–Bertani agar plates, in triplicates, to enumerate the bacterial count, and another 1,000 µl was used for DNA preparation.

For each primer set, the colony-forming units (CFUs) per LAMP reaction were calculated, using the following formula:

$$\mathrm{CFUs per LAMP reaction:}\frac{\left(\mathrm{Volume of DNA template}\right)\text{∗}\left(\mathrm{CFUs before DNA extraction}\right)}{\left(\mathrm{Volume of buffer solution used for DNA elution}\right)}$$

### LAMP Assays and Measures of Test Accuracy

LAMP primers were designed using Optigene LAMP Designer software. For each *K. pneumoniae* and carbapenemase target gene, a minimum of two sets of four to six primers; outer primers (F3 and B3), inner primers (FIP and BIP), and for some sets loop primers (LoopF and/or LoopB), recognizing six to eight distinct regions of target DNA, were designed (Table 1).

**TABLE 1:** LAMP primer sets designed for this study, targeting the *K. pneumoniae* Inner membrane protein (*yhaI*) and carbapenemase genes (*bla*_KPC_, *bla*_NDM_, *bla*_OXA-48-like_, *bla*_IMP_, *and bla*_VIM_).

Genes^a^	Primer	Sequence (5′ to 3′)
*yhaI*	*yhaI*-F3	ATGTATTGATCGCCACCG
	*yhaI*-B3	GAG​CCA​GCG​AAA​TAA​TTG​C
	*yhaI*-FIP	TCA​ACA​GCA​GCC​AGA​GCG​CTC​AAT​ACG​GCA​TCC​TCA​G
	*yhaI*-BIP	CGG​CGG​ATG​CAT​GAT​ATC​GGA​AAC​CAC​GGA​ATG​ATA​ACC​C
	*yhaI*-LF	GGC​AAA​GGG​AAT​GAC​AAC​AAA
	*yhaI*-LB	CCGGGATCTGGTTTGTGT
*bla*_KPC_	KPC-F3	CGT​GAC​GGA​AAG​CTT​ACA​A
	KPC-B3	GCTGTGCTTGTCATCCTT
	KPC-FIP	GCC​GGT​CGT​GTT​TCC​CTT​AAA​CTG​ACA​CTG​GGC​TCT
	KPC-BIP	ACT​GGG​CAG​TCG​GAG​ACA​AGA​CGA​CGG​CAT​AGT​CAT
	KPC-LF	AGC​CAA​TCA​ACA​AAC​TGC​TG
	KPC-LB	GAGTGTATGGCACGGCAA
*bla*_NDM_	NDM-F3	CAT​TAG​CCG​CTG​CAT​TGA​TG
	NDM-B3	CCGCCATCCCTGACGATC
	NDM-FIP	ATC​GCC​AAA​CCG​TTG​GTC​GCC​CGG​TGA​AAT​CCG​CCC​G
	NDM-BIP	TGG​TTT​TCC​GCC​AGC​TCG​CAG​CGA​CTG​CCC​CGA​AAC
	NDM-LF	TCC​ATT​TGC​TGG​CCA​ATC​G
	NDM-LB	ACC​GAA​TGT​CTG​GCA​GCA​CA
*bla*_OXA-48-like_	OXA48-like-F3	ATCACAGGGCGTAGTTGT
	OXA48-like-B3	CGTCTGTCCATCCCACTT
	OXA48-like-FIP	TGC​TTG​GTT​CGC​CCG​TTT​AAC​TCT​GGA​ATG​AGA​ATA​AGC​AG
	OXA48-like-BIP	TAC​CCG​CAT​CTA​CCT​TTA​AAA​TTC​CAA​GAC​TTG​GTG​TTC​ATC​CT
	OXA48-like-LB	GCT​TGA​TCG​CCC​TCG​ATT​TG
*bla*_IMP_	IMP-F3	CACTTGGTTTGTGGAACG
	IMP-B3	AGCCAATAGTTAACCCCG
	IMP-FIP	TAA​GCC​ACT​CTA​TTC​CGC​CCA​AAA​TAA​AAG​GCA​GTA​TTT​CCT​CTC
	IMP-BIP	CAT​CCC​CAC​GTA​TGC​GTC​TGC​CAA​ATG​AAT​TTT​TAG​CTT​GAA​CC
	IMP-LB	CTA​ATG​AGC​TGC​TTA​AAA​AAG​ACG​G
*bla*_VIM_	VIM-F3	AATTCCGGTCGGAGAGGT
	VIM-B3	AAAGTGCGTGGAGACTGC
	VIM-FIP	CAT​TGG​ACG​GGT​AGA​CCG​CGC​CAG​ATT​GCC​GAT​GGT​GT
	VIM-BIP	TGA​TTG​ATA​CAG​CGT​GGG​GTG​CAC​GGG​AAG​TCC​AAT​TTG​CT
	VIM-LF	CGT​TGC​GAT​ATG​CGA​CCA​A

a The complete coding sequences of *yhaI* (Kp00840_02010 in Supplementary List S6); *bla*_KPC_ (NC_023904.1), *bla*_NDM-1_ (FN396876), *bla*_OXA-48_ (AY236073), *bla*_IMP-4_ (AF244145), and *bla*_VIM-1_ (Y18050) were the reference sequences.

LAMP was initially performed using the fluorometric detection method. Individual LAMP reactions consisted of 15 µl of ISO-004 Isothermal Mastermix (OptiGene, Ltd., United Kingdom), 2.5 µl of 10× primer mix containing 8 µM of FIP/BIP, 2 µM of F3/B3 and 4 µM of LoopF/LoopB, 2.5 µl of nuclease-free water and 5 µl of DNA template, or nuclease-free water for negative controls. LAMP reactions were run at 65°C for 30 min, and the fluorescence of a DNA binding dye was evaluated every 15 s to generate amplification curves, using Genie II device (OptiGene, Ltd.). Amplicons’ annealing profiling was also performed by a temperature gradient from 98°C to 80°C at a rate of 0.05°C × s^−1^, using Genie II. Optimal primers for *K. pneumoniae* detection and differentiation were chosen based on accuracy and speed of amplification.

Once the best primers were chosen, LAMP was also performed using a colorimetric method of detection. Individual LAMP reactions consisted of 12.5 µl of WarmStart^®^ Colorimetric LAMP 2× Mastermix (New England Biolabs Ltd., United Kingdom), 2.5 µl of 10× primers mix containing 16 μM FIP/BIP, 2 μM F3/B3 and 4 μM LoopF/LoopB, 5 µl of nuclease-free water and 5 µl of DNA template, or nuclease-free water for negative controls. Successful LAMP amplification induced a pH change, resulting from proton accumulation due to dNTP incorporation. This pH acidification was detected by phenol red, a pH-sensitive dye, which color changed from pink (negative) to yellow (positive). Photographs of LAMP reactions were taken after 30 min of amplification at 65°C with a MyBlock™ mini dry bath (Benchmark Scientific, United States).

For each primer set, contingency tables were constructed, and sensitivity, specificity, and positive and negative predictive values were calculated.

### Testing of Clinical Sputum Samples

Clinical sputum samples were collected into a sterile sealed container according to routine procedure at Ruijin Hospital. A total of 40 clinical sputum samples (1–40) were selected for this study. According to culture and MALDI-TOF MS, 18 samples were positive for *K. pneumoniae*, and 22 samples were negative (Supplementary Table S7). Among the 22 negative samples, 17 contained other bacterial pathogens (Supplementary Table S7). All the sputum samples were decontaminated by adding an equal volume of 10% NaOH solution, vortexed thoroughly, and incubated at room temperature for 5 min. Following incubation, 400 μl of each sample was centrifuged for 5 min at 12,000 rpm at ambient temperature. Following centrifugation, the supernatants were discarded, and the pellets were used for DNA extraction.

Susceptibility to imipenem and meropenem was determined in the *K. pneumoniae* isolates recovered from the sputum samples, using the Vitek^®^ 2 (bioMérieux, Inc.) automated system. Minimum inhibitory concentrations were classified according to breakpoints established by the Clinical and Laboratory Standards Institute (Wayne, 2020).

To assess the feasibility of blood samples testing with our diagnostics, the limit of bacterial copies per LAMP reaction was also determined in spiked defibrinated sheep blood samples, using a protocol previously described in other studies (Goto et al., 2010; Manajit et al., 2018). For these studies, a 16-h broth culture of *K. pneumoniae* HS11286 (OD600 nm at 0.5) was serially diluted 10-fold dilutions in 0.85% of normal saline; 100 µl of each dilution was plated on Luria–Bertani agar plates, in triplicates, to enumerate the bacterial count. Then, 20 µl of 10 bacterial cell suspensions, with concentrations ranging from of 0.38 CFUs/ml to 3.8 × 10^7^ CFUs/ml, were used to spike 180 µl sterile defibrinated sheep blood samples each. All 10 spiked blood samples (200 µl each) were used for DNA extraction, and DNA was eluted in 100 µl. For each primer set, the CFUs per LAMP reaction were calculated (using the same formula than described in 2.4).

### PCR Verification of the LAMP Assays

DNA extracted from the isolates and clinical samples was tested for the presence of the carbapenemase genes *bla*_KPC_, *bla*_NDM_, *bla*_OXA-48-like_, *bla*_IMP_, and *bla*_VIM_ and of the *yhaI* gene by PCR. The PCR primers for *yhaI* (249bp size product) and *bla*_KPC_ (240bp size product) genes were designed for this study using the Primer-Blast software (Ye et al., 2012) (Table 2). The PCR testing for the carbapenemase genes *bla*_NDM_ *, bla*_OXA-48-like_ *, bla*_IMP_ *,* and *bla*_VIM_ was performed with specific primers and conditions as previously described (Dallenne et al., 2010; Han et al., 2020) (Table 2).

**TABLE 2:** PCR primers for the *K. pneumoniae* inner membrane protein (*yhaI*) and carbapenemase genes (*bla*_KPC_, *bla*_NDM_, *bla*_OXA-48-like_, *bla*_IMP_, and *bla*_VIM_).

Genes	Primer^a^	Sequence^b^ (5′ to 3′)	Product size (bp)
*yhaI*	*yhaI*-F	ATT​TGA​GCG​GCT​GGA​AAG​AG	249
	*yhaI*-R	AGC​GGC​CGA​TAT​CAT​GCA​T	
*bla*_KPC_	KPC-F	TTG​TTG​CTG​AAG​GAG​TTG​GG	240
	KPC-R	GGT​CGT​GTT​TCC​CTT​TAG​CC	
*bla*_NDM_	NDM-F	GGT​TTG​GCG​ATC​TGG​TTT​TC	621
	NDM-R	CGG​AAT​GGC​TCA​TCA​CGA​TC	
*bla*_OXA-48-like_	OXA-48like-F	GCG​TGG​TTA​AGG​ATG​AAC​AC	438
	OXA-48like-R	CAT​CAA​GTT​CAA​CCC​AAC​CG	
*bla*_IMP_	IMP-F	GGAATAGAGTGGCTTAAYTCTC	232
	IMP-R	GGTTTAAYAAAACAACCACC	
*bla*_VIM_	VIM-F	GAT​GGT​GTT​TGG​TCG​CAT​A	390
	VIM-R	CGAATGCGCAGCACCAG	

a The PCR primers of carbapenemase genes (*bla*_KPC_, *bla*_NDM_, *bla*_OXA-48-like_, *bla*_IMP_, and *bla*_VIM_) used were those as previously described by Dallenne et al. (2010), Han et al. (2020), respectively;

^b^ *Y* = *C* or *T*.

## Results

### Identification of Gene Targets for the Detection of *K. pneumoniae*

**FIGURE 1:**
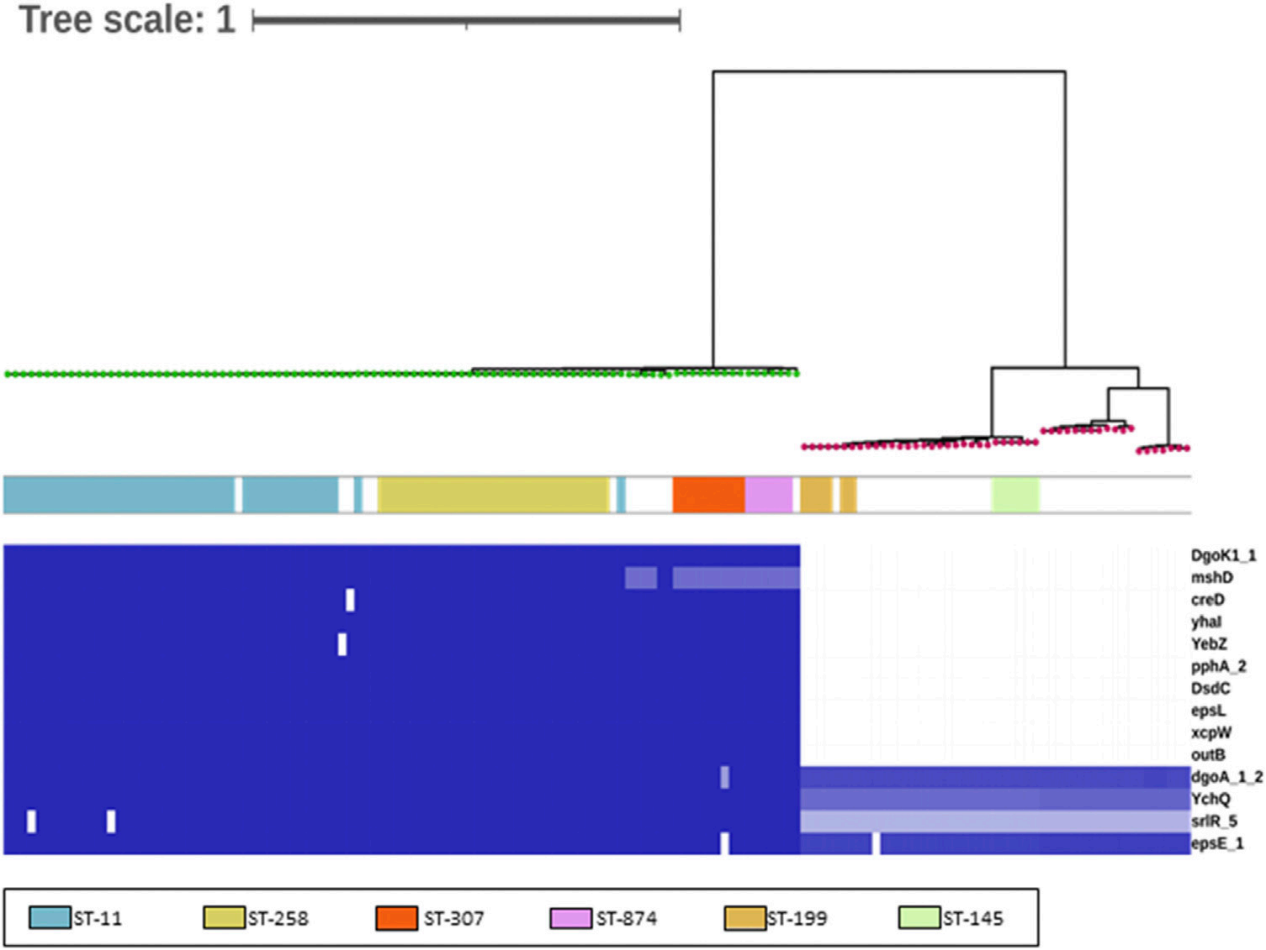
Identification of discriminatory gene targets for *K. pneumoniae*. This matrix was generated using BLAST (Abricate) using an 80% identity and coverage threshold to identify potential variation. Intensity of the blue heat map indicates identity relative to query sequences (Supplementary File S2) (blue = 100%, white = 0%/absence). Genes encoded by both *Klebsiella* species are also listed, for reference (bottom 4). The genomes are ordered according to the branching produced using core genome SNP as identified using ParSNP and FastTree. The green nodes are *K. pneumoniae* genomes, and the red nodes, *K. oxytoca* genomes. The colors indicate the six sequence types (STs) identified in the *Klebsiella* included in the study.

A total of 50 *K. pneumoniae* genomes from Europe, 50 *K. pneumoniae* genomes from China (Supplementary Table S4), and 50 *K. oxytoca* genomes (Supplementary Table S5) were selected to identify unique targets for *K. pneumoniae*. After annotation of genomes assemblies using Prokka and construction of the pan-genomes using Roary, the identification of genes specifically associated with *K. pneumoniae* was performed using Scoary. A total of nine genes were associated with *K. pneumoniae* at a frequency of 100% and 0% with *K. oxytoca* in the studied *Klebsiella* genomes (Table 3; Figure 1). These nine genes were incorporated into a custom ABRicate (Seemann, 2019) database (Supplementary List S6) and used to screen a large collection of 7,320 *K. pneumoniae* genomes derived from all over the world (Supplementary Table S3), using cutoffs of 80% identity and 80% coverage. Out of the nine genes, seven were found to be universally present in the 7,320 *K. pneumoniae* genomes (Table 3, Supplementary Table S8). NCBI BLAST searches, excluding *K. pneumoniae*, were then performed and revealed that three genes were exclusive to *K. pneumoniae* (Table 3; Figure 1). These three genes were *yhaI* (Kp00840_02010 in Supplementary List S6), encoding an inner membrane protein; *xcpW* (Kp00844_46720 in Supplementary List S6), encoding the type II secretion system protein J; and *epsL* (Kp00844_46740 in Supplementary List S6), encoding the type II secretion system protein L. These genes were selected as putative targets for design of a LAMP assay to rapidly detect *K. pneumoniae* in clinical samples.

**TABLE 3:** List of gene targets identified for the detection of *K. pneumoniae*.

Gene name	Protein	Sequence
***yhaI***	**Inner membrane protein**	**Kp00840_02010**
***epsL***	**Type II secretion system protein L**	**Kp00844_46720**
***xcpW***	**Type II secretion system protein J**	**Kp00844_46740**
***outB***	**General secretion pathway protein B**	**Kp00844_46830**
***creD***	**Inner membrane protein**	**Kp00840_01750**
***YebZ***	**Inner membrane protein**	**Kp00844_07280**
***pphA_2***	**Serine/threonine-protein phosphatase**	**Kp00844_07320**
*DgoK1_1*	Putative 2-dehydro-3-deoxygalactonokinase	Kp00840_01120
*DsdC*	d-Serine deaminase activator	Kp00844_23880

The Scoary analyses output a list of nine genes associated with *K. pneumoniae* at a frequency of 100 and 0% with *K. oxytoca*. Of the nine genes, seven (genes in bold) were found in the 7,320 *K. pneumoniae* genomes, using a custom ABRicate. NCBI BLAST searches identified three genes (in bold and underlined) as gene targets.

### Specificity, Sensitivity, and Limit of Detection of the *K. pneumoniae* LAMP Assay on Clinical Isolates

**FIGURE 2:**
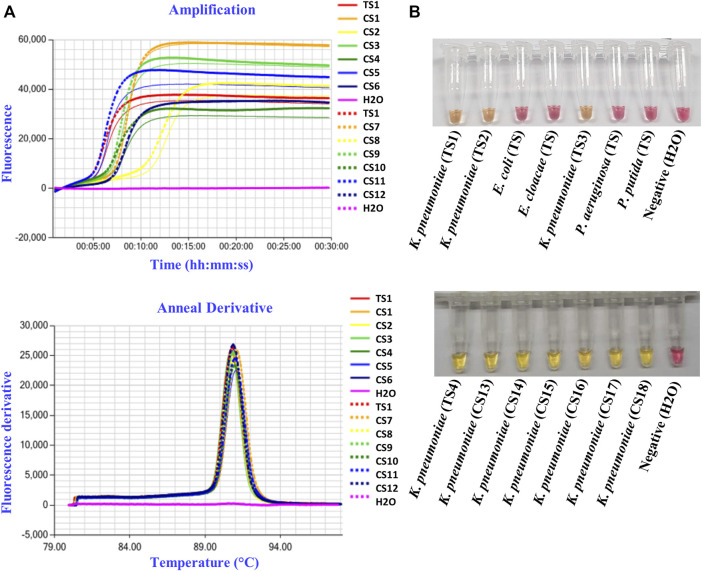
*K. pneumoniae* LAMP detection in pure cultures. **(A)** Representative fluorometric LAMP results, targeting the *yhaI* gene, performed on type strain (TS1) and clinical isolates (CS1–12) of *K. pneumoniae* from the United Kingdom, with H_2_O as a negative control. **(B)** Representative colorimetric LAMP results, targeting the *yhaI* gene, performed on type strains (TS) and clinical isolates (CS) of *K. pneumoniae* from the United Kingdom (TS1–3) and from China (TS4 and CS13–18), as well as on type strains of *E. coli*, *E. cloacae*, *Pseudomonas aeruginosa*, and *P. putida*, with H_2_O as a negative control.

To detect *K. pneumoniae*, LAMP primers were designed for amplification of the three target genes identified using comparative genomics: *yhaI*, *epsL*, and *xcpW*. Each primer set was used to test a panel of 15 *K. pneumoniae* strains, with a fluorometric LAMP detection method (Genie II; Optigene Ltd., Horsham, England). Following screening on a panel of strains, the *yhaI* gene primer set that showed the best accuracy and speed of amplification was selected and subsequently used to amplify the genomic DNA of 45 *K. pneumoniae* strains from the United Kingdom, which it did in less than 15 min. The amplification of a single product in the LAMP reactions was confirmed, by analyzing the melting curves (Figure 2 and Table 4). A single sharp dissociation peak was observed for the *yhaI* gene primer set only in the LAMP reactions containing *K. pneumoniae* templates. The annealing temperature was 90.5°C ± 1°C. LAMP detection of *K. pneumoniae* was also evaluated with colorimetric detection, for use as a point-of-care diagnostic requiring minimal equipment. Using this detection method and the *yhaI* gene primers, a 15-min LAMP reaction was determined to be sufficient for the accurate detection of *K. pneumoniae* (Figure 2 and Table 4). Using colorimetric LAMP, 3 *K. pneumoniae* type strains, 47 *K. pneumoniae* clinical isolates from United Kingdom, and 2 *K. pneumoniae* type strains and 120 *K. pneumoniae* clinical isolates from China were detected within 15 min. No amplification and no annealing or color change were observed in the no-template control reactions, irrespective of the method of detection.

**TABLE 4:** Summary of *K. pneumoniae*–specific LAMP results.

Tested isolates	*yhaI* detection with colorimetric method (on 319 isolates)	*yhaI* detection with fluorometric method (on 88 isolates)
*K pneumoniae* type strains (PHE and ATCC)	5/5 positives in 15 min	5/5 positives in 10 min
United Kingdom *K. pneumoniae* clinical isolates (partner hospitals)	47/47 positives in 15 min	47/47 positives in 13 min
Ruijin Hospital *K. pneumoniae* clinical isolates	120/120 in 15 min	
*K. aerogenes* (China)	0/5 clinical isolates	
*K. oxytoca* (United Kingdom and China)	0/9 type strains and 0/5 clinical isolates	0/9 type strains
*E. coli* (United Kingdom and China)	0/4 type strains and 0/20 clinical isolates	0/4 type strains
*S. enterica* (United Kingdom and China)	0/5 type strains and 0/25 clinical isolates	0/5 type strains and 0/5 clinical isolates
*Acinetobacter* spp. (United Kingdom and China)	0/2 type strains and 0/20 clinical isolates	0/2 type strains
*Pseudomonas* spp. (United Kingdom and China)	0/3 type strains and 0/20 clinical isolates	0/2 type strains
*S. aureus* (United Kingdom and China)	0/5 type strains and 0/20 clinical isolates	0/5 type strains
*E. cloacae* (United Kingdom)	0/2 positive	0/2 type strains
*Y. enterocolitica* (United Kingdom)	0/1 positive	0/1 type strains
*S. pyogenes* (United Kingdom)	0/1 positive	0/1 type strains

All results obtained by LAMP targeting the *yhaI* gene, performed on 319 strains: 5 *K. pneumoniae* type strains, 167 *K. pneumoniae* clinical isolates, 32 non–*K. pneumoniae* type strains, and 115 non–*K. pneumoniae* clinical isolates.

BLAST queries of the primer binding regions from *yhaI* primer set against the NCBI nonredundant database, excluding *K. pneumoniae*, demonstrated extremely low sequence identity. Subsequent testing of the primer set on 10 other bacterial species (*K. aerogenes*, *K. oxytoca*, *E. coli*, *S. enterica*, *Acinetobacter* spp., *Pseudomonas* spp., *S. aureus*, *E. cloacae*, *Y. enterocolitica*, and *S. pyogenes*) showed no cross reactions (Table 4).

For *yhaI* gene detection, the calculated sensitivity, specificity, and positive and negative predictive values (Table 5, established using LAMP results obtained on the 319 strains described in Table 4) were all 100% on pure cultures.

**TABLE 5:** Contingency table for *K. pneumoniae*–specific LAMP.

LAMP test results	Reference method (MALDI-TOF and culture)		Total
	Positive	Negative	
Positive	172	0	172
Negative	0	147	147
Total	172	147	319

Results obtained by LAMP targeting the *yhaI* gene, performed on 319 strains: 5 *K. pneumoniae* type strains, 167 *K. pneumoniae* clinical isolates, 32 non–*K. pneumoniae* type strains, and 115 non–*K. pneumoniae* clinical isolates.

To determine the limit of detection of the designed LAMP assay, the genomic DNAs of the type strains *K. pneumoniae* NCTC13809 and *K. pneumoniae* HS11286 were extracted and then diluted from 10 ng/μL to 1 fg/μl. Using fluorometric detection, the lowest concentration of *K. pneumoniae* NCTC13809 DNA amplified by the *yhaI* primers was 100 fg/μl (equivalent to 16.1 gene copies/µl). Using colorimetric detection, the limit of detection for *yhaI* in *K. pneumoniae* HS11286 was 10 pg/reaction, which is 100 times lower than the routine PCR method used at the Ruijin Hospital. When considering the viable CFU counts, used for DNA template preparation, the limit of detection for *yhaI* was found to be 3.8 CFUs/reaction.

### LAMP Assays to Detect Carbapenem Resistance in Clinical Isolates

**FIGURE 3:**
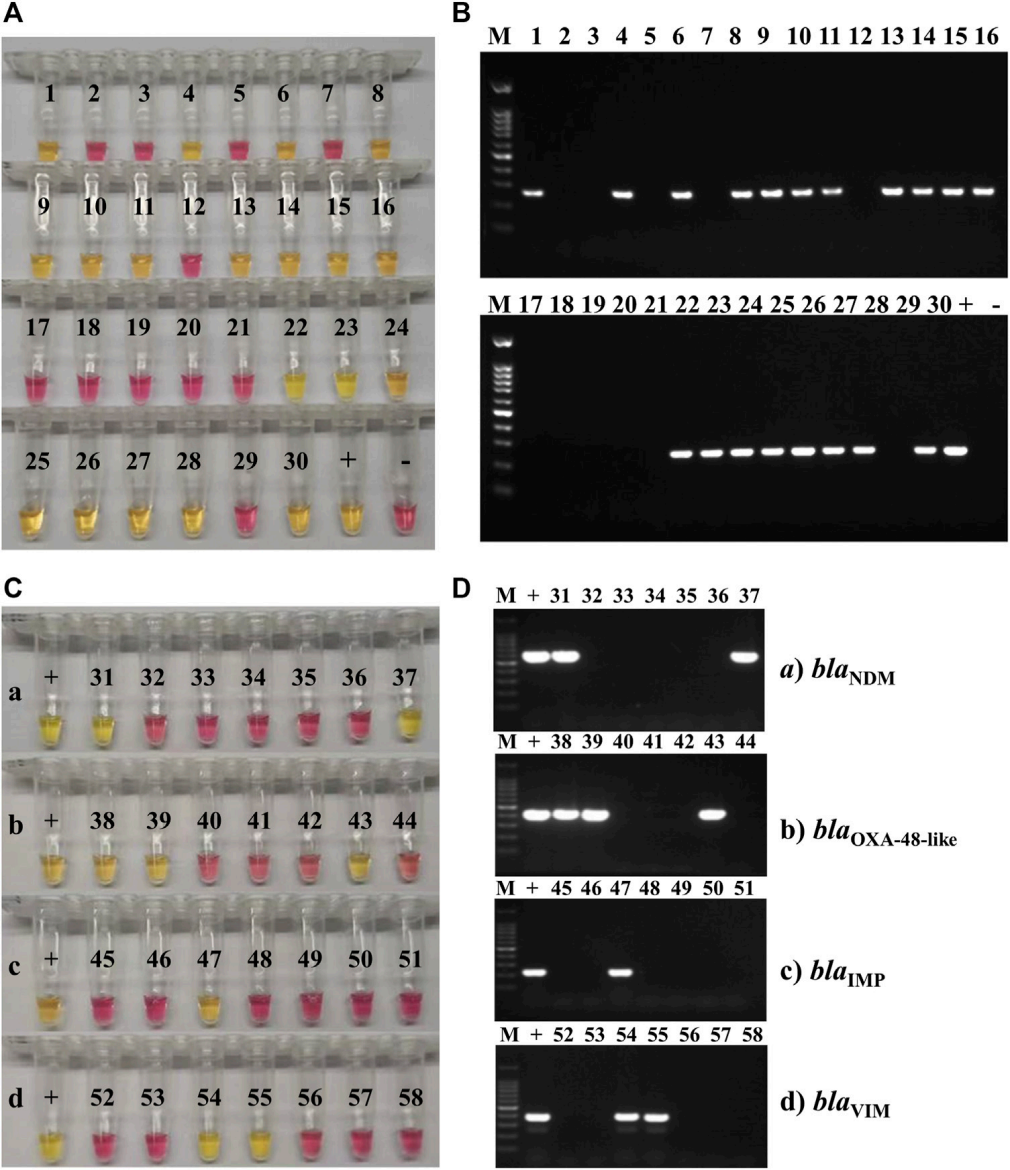
Carbapenemase-producing genes LAMP and PCR testing on clinical isolates. **(A)** Representative colorimetric LAMP results targeting the *bla*_KPC_ gene performed on 30 of the 120 clinical isolates of *K. pneumoniae* collected at Ruijin Hospital (Shanghai, China); 19 isolates were *bla*_KPC_-positive (yellow; 1, 4, 6, 8–11, 13–16, 22–28, and 30), and 11 were negative (pink). **(B)** Representative results of endpoint PCR targeting the *bla*_KPC_ gene performed on the same 30 clinical isolates of *K. pneumoniae* than the ones tested by LAMP. Nineteen isolates were *bla*_KPC_-positive (amplification band at 240 bp; 1, 4, 6, 8–11, 13–16, 22–28, and 30), and 11 were negative (no amplification band). The marker (M) used for these experiments was a 100-bp DNA ladder. **(C)** Representative colorimetric LAMP results targeting the *bla*_NDM_ (a), *bla*_OXA-48-like_ (b), *bla*_IMP_ (c), and *bla*_VIM_, (d) carbapenemase-producing genes, obtained on 28 of the 120 clinical isolates of *K. pneumoniae* collected at Ruijin Hospital (Shanghai, China). For each AMR gene, the positive control (+) was a clinical isolate, collected from the Ruijin Hospital (Shanghai, China) and confirmed to be positive by both PCR and sequencing. Two *K. pneumoniae* clinical isolates were positive (yellow; 31 and 37) for *bla*_NDM_ (a), three were positive (yellow; 38, 39, and 43) for *bla*_OXA-48-like_ (b), one was positive (yellow; 47) for *bla*_IMP_ (c), and two were positive (yellow; 54 and 55) for *bla*_VIM_ (d) genes. **(D)** Representative PCR amplification results targeting the *bla*_NDM_ (a), *bla*_OXA-48-like_ (b), *bla*_IMP_ (c), and *bla*_VIM_ (d) genes, obtained on the same 28 clinical isolates of *K. pneumoniae* than the ones tested by LAMP. For each AMR gene, the positive control (+) was a clinical isolate, collected from the Ruijin Hospital (Shanghai, China) and confirmed to be positive by both PCR and sequencing. Two *K. pneumoniae* clinical isolates were positive (amplification band at 621 bp; 31 and 37) for *bla*_NDM_ (a), three were positive (amplification band at 438 bp; 38, 39 and 43) for *bla*_OXA-48-like_ (b), one was positive (amplification band at 232 bp; 47) for *bla*_IMP_ (c), and two were positive (amplification band at 390 bp; 54 and 55) for *bla*_VIM_ (d) genes. The marker (M) used for these experiments was a 100-bp DNA ladder.

For the detection of carbapenemase (*bla*_*KPC*_)-producing *K. pneumoniae*, the set of LAMP primers that showed the best accuracy and speed of amplification was validated on a panel of 17 *K. pneumoniae* and *K. oxytoca* carbapenem-resistant strains from the United Kingdom. All 17 strains were *bla*_*KPC*_-positive using both fluorometric LAMP and *bla*_*KPC*_ PCR detection. For LAMP, a single sharp dissociation peak was observed only in the LAMP reactions containing *bla*_*KPC*_-positive templates, at an annealing temperature of 92.5°C ± 0.5°C. For PCR, a single band was observed (approximately 240 bp). *bla*_KPC_ primers were subsequently tested on *K. pneumoniae* type strain HS11286 and clinical isolates collected at Ruijin Hospital (Shanghai, China). *K. pneumoniae* type strain HS11286 and 66 of the 120 *K. pneumoniae* clinical isolates were *bla*_KPC_-positive within 15 min (Figure 3; Table 6), using colorimetric LAMP. The strains that were *bla*_KPC_-positive by LAMP were also positive by PCR and were carbapenem-resistant according to AST using Vitek^®^ 2 automated system. No amplification and no annealing or color change were observed in the no-template control reactions, irrespective of the method of detection (Figure 3; Table 6). In addition, all the strains *bla*_KPC_ negatives by PCR were also negative by *bla*_KPC_ LAMP. For *bla*_*KPC*_ gene detection, the calculated sensitivity, specificity, and positive and negative values (Table 6) were all 100%.

**TABLE 6:** Summary of carbapenem resistance–specific LAMP and PCR results.

LAMP test results	Reference method (PCR)		Total
	Positive	Negative	
Positive	68	0	68
Negative	0	57	57
Total	68	57	125

Contingency table displaying LAMP and PCR results targeting the *bla*_*KPC*_ gene performed on 125 *K. pneumoniae* strains; 68 *K. pneumoniae* strains were *bla*_*KPC*_-positive by both LAMP and PCR, and 57 strains were negative by both LAMP and PCR.

To determine the limit of detection of the *bla*_*KPC*_ LAMP, the genomic DNAs from the type strains *K. pneumoniae* NCTC13809 and *K. pneumoniae* HS11286 (both *bla*_*KPC*_-positive) were extracted and then diluted from 10 ng/μl to 1 fg/μl. The lowest concentration of *K. pneumoniae* NCTC13809 and HS11286 DNA amplified by the *bla*_*KPC*_ primers was 10 pg/μl (equivalent to 1,609.2 gene copies/µl), using both fluorometric and colorimetric detection. When considering the viable CFU counts, used for DNA template preparation, the limit of detection was found to be 3.8 CFUs/reaction.

For the detection of other carbapenem-resistance genes *bla*_NDM_, *bla*_OXA-48-like_, *bla*_IMP_, and *bla*_VIM_, LAMP primers were designed and tested on *K. pneumoniae* or other species of clinical isolates from China. All clinical isolates were also tested by PCR for the *bla*_NDM_, *bla*_OXA-48-like_, *bla*_IMP_, and *bla*_VIM_ genes (Figure 3). Seven of 18 isolates were *bla*_NDM_-positive, 7 of 18 isolates were *bla*_OXA-48-like_-positive, 3 of 18 isolates were *bla*_IMP_-positive, and 4 of 18 isolates were *bla*_VIM_-positive, using both colorimetric LAMP and PCR. The strains that were positive by both LAMP and PCR were also carbapenem-resistant according to AST using Vitek^®^ 2 automated system.

### Testing of Clinical Sputum Samples and Spiked Blood

**FIGURE 4:**
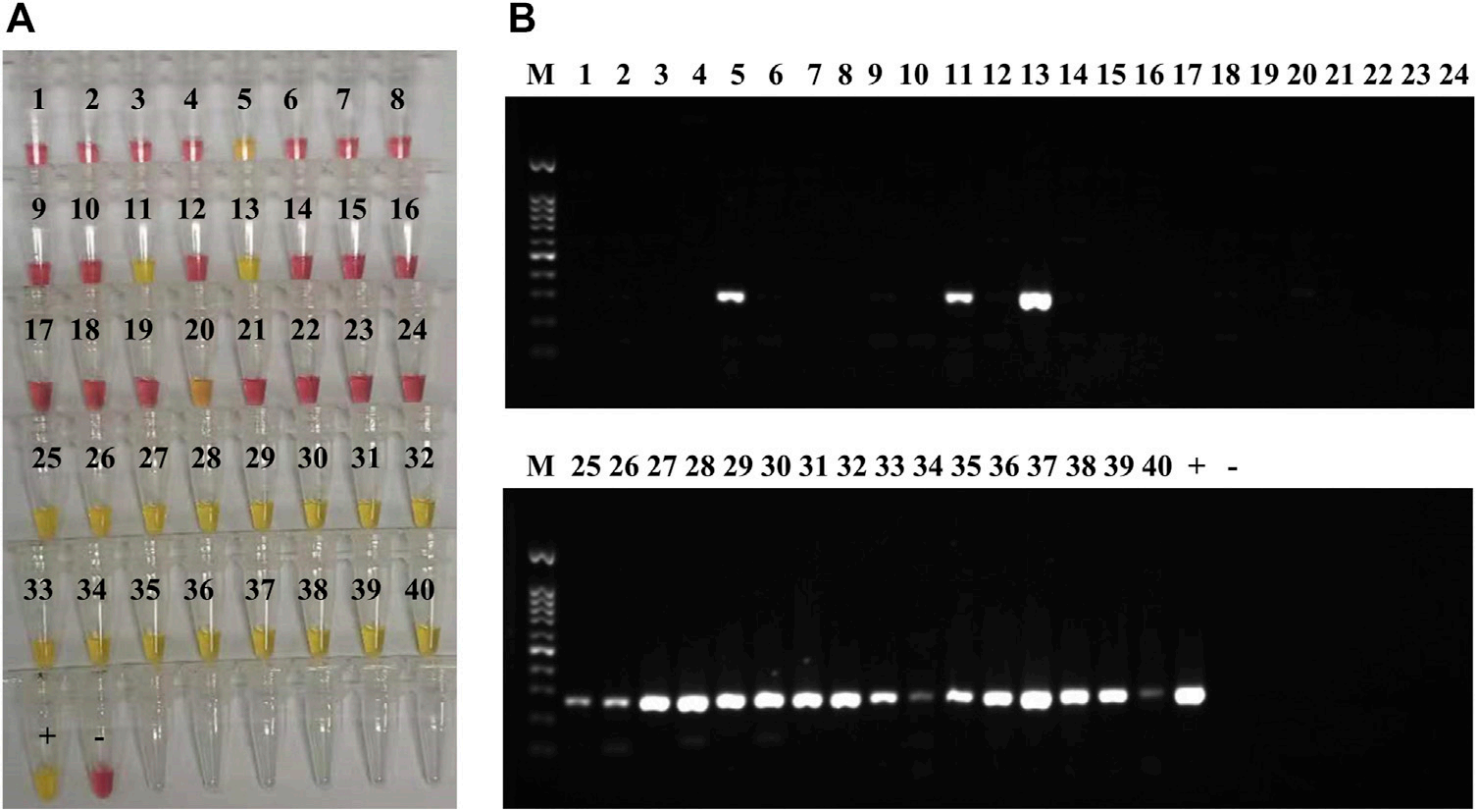
*K. pneumoniae* LAMP and PCR detection in clinical sputum samples. **(A)** Colorimetric LAMP results, targeting the *yhaI* gene, obtained on DNA extracted from 40 clinical sputum samples; 20 of the samples were positive (yellow; 5, 11, 13, 20, and 25–40), and 20 were negative (pink; 1–4, 6–10, 12, 14–19, and 21–24). DNA extracted from *K. pneumoniae* HS11286 was used as positive control (+), and the negative control (−) was molecular H_2_O. **(B)** PCR results, for *yhaI* gene amplification, obtained on DNA extracted 40 clinical sputum samples (same than A); 19 of the samples were positive (amplification bands; 5, 11, 13, and 25–40), and 21 were negative (no band; 1–4, 6–10, 12, and 14–24). DNA extracted from *K. pneumoniae* HS11286 was used as positive control (+) and the negative control (−) was molecular H_2_O. The marker (M) was a 100-bp DNA ladder.

According to MALDI-TOF MS and culture, 18 of the clinical sputum samples were positive for *K. pneumoniae* (11, 13, 25–40; Supplementary Table S7), and 22 samples were negative for Kp (1–10, 12, and 14–24; Supplementary Table S7). Among the 22 Kp-negative samples, 17 contained other bacterial pathogens (Supplementary Table S7). All the 18 samples positive for Kp by culture and MALDI-TOF were also positive by LAMP and PCR assays targeting the *yhaI* gene (11, 13, 25–40; Figure 4 and Supplementary Table S7). Of the 22 samples negative for Kp by culture and MALDI-TOF, 20 were also negative by LAMP (1–4, 6–10, 12, 14–19, and 21–24; Figure 4 and Supplementary Table S7), and 21 were negative by PCR (1–4, 6–10, 12, 14–24; Figure 4 and Supplementary Table S7). According to MALDI-TOF, sample 5 contained only *P. aeruginosa*, whereas it was positive by both LAMP and PCR for the *yhaI* gene (Figure 4 and Supplementary Table S7). Sample 20, containing *Serratia marcescens* and *P. aeruginosa* according to MALDI-TOF, was slightly positive by LAMP and negative by PCR (Figure 4 and Supplementary Table S7). The calculated sensitivity, specificity, and positive and negative predictive values of the Kp LAMP assay (*yhaI* gene) were 100%, 91%, and 90% and 100%, respectively, when tested on clinical sputum samples, in comparison with culture and MALDI-TOF methods.

**FIGURE 5:**
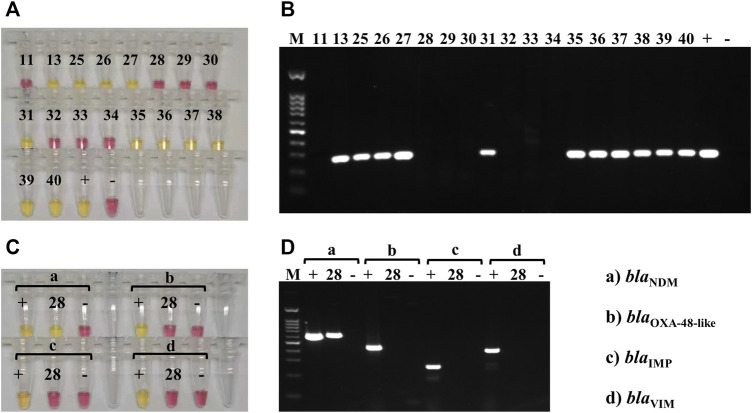
Carbapenemase-producing genes LAMP and PCR testing on clinical sputum samples. **(A)** Colorimetric LAMP results, targeting the *bla*_KPC_ gene, obtained on DNA extracted from the 18 clinical sputum samples that contained *K*. *pneumoniae* (according to MALDI-TOF); 11 of the samples were *bla*_*KPC*_-positive (yellow; 13, 25–27, 31, and 35–40), and 7 were negative (pink). DNA extracted from *K. pneumoniae* HS11286 was used as a positive control (+), and the negative control (−) was molecular-grade H_2_O. **(B)** PCR results, for *bla*_KPC_ amplification, obtained on DNA extracted from the 18 clinical sputum samples that contained *K*. *pneumoniae* (same as A); 11 of the samples were *bla*_*KPC*_-positive (amplification bands; 13, 25–27, 31, and 35–40), and 7 were negative (no band). DNA extracted from *K. pneumoniae* HS11286 was used as positive control (+), and the negative control (−) was molecular-grade H_2_O. The marker (M) was a 100-bp DNA ladder. **(C)** Colorimetric LAMP results, targeting the *bla*_*NDM*_ (a), *bla*_*OXA-48like*_ (b), *bla*_*IMP*_ (c), and *bla*_*VIM*_ (d) genes, obtained on the DNA extracted from sample 28. Positive controls (+) and negative controls (−) were used for each assay. Sample 28 was positive (yellow) for *bla*_*NDM*_ (a) and negative (pink) for the other carbapenemase-producing genes (b–d). **(D)** PCR results, targeting the *bla*_*NDM*_ (a), *bla*_*OXA-48like*_ (b), *bla*_*IMP*_ (c), and *bla*_*VIM*_ (d) genes, obtained on the DNA extracted from sample 28. Positive controls (+) and negative controls (−) were used for each assay. Sample 28 was positive (amplification band at 621 bp) for *bla*_*NDM*_ (a) and negative (no band) for the other carbapenemase-producing genes. The marker (M) was a 100-bp DNA ladder.

Susceptibility tests to imipenem and meropenem (using the Vitek^®^ 2 automated system; bioMérieux, Inc.) were performed on the 18 *K. pneumoniae* isolates from sputum samples and showed that 12 of the isolates were resistant to these carbapenems (13, 25–28, 31, and 35–40; Supplementary Table S7). Both LAMP and PCR assays, performed on the DNA directly extracted from the sputum samples, showed that 11 of 12 carbapenem-resistant *K. pneumoniae* isolates carried the *bla*_*KPC*_ gene (13, 25–27, 31 and 35–40; Figure 5 and Supplementary Table S7) and that one isolate harbored the *bla*_*NDM*_ gene (28; Figure 5 and Supplementary Table S7).

To assess the feasibility of blood samples testing with our diagnostics, the limit of bacterial copies per LAMP reaction was also determined in spiked defibrinated sheep blood samples. A total of 10 bacterial cell suspensions, with concentrations ranging from of 0.38 CFUs/ml to 3.8 × 10^7^ CFUs/ml, were used to spike sterile defibrinated sheep blood samples that were then extracted using a commercial DNA extraction kit. The minimal detectable concentration for LAMP was 7.6 CFUs/reaction for both *yhaI* and *bla*_KPC_ detection in colorimetric LAMP.

## Discussion

Public health systems around the world would greatly benefit from an accurate, rapid, and user-friendly method to diagnose pathogenic microorganisms and their potential AMR. In this study, we identified specific markers for the rapid and accurate detection of *K. pneumoniae*, using comparative genomics tools. These markers were found in more than 7,000 *K. pneumoniae* genomes from all over the world and absent from other species of *Klebsiella* and other bacterial genera; they were then chosen as target genes for *K. pneumoniae*–specific LAMP diagnostics. We also developed LAMP assays to rapidly and accurately detect the most common carbapenemase-producing genes: *bla*_KPC_, *bla*_NDM_, *bla*_OXA-48-like_, *bla*_IMP_, and *bla*_VIM_.

The LAMP assays developed herein can achieve sensitive and accurate detection of *K. pneumoniae* and carbapenemase genes, with performance comparable to gold-standard clinical methodologies that are time-consuming or based on expensive specialized instruments. Indeed, when compared with culture-based methods or PCR, LAMP results achieved sensitivity, specificity, and positive and negative values of 100% in both pure cultures and spiked blood samples. In clinical sputum samples, the calculated sensitivity, specificity, and positive and negative values of the *yhaI* LAMP (*K. pneumoniae* detection) were 100%, 91%, and 90% and 100%, respectively, when compared with MALDI-TOF MS and culture. Only 2 clinical samples out of 40 were *yhaI*-positive by LAMP, although not identified as containing *K. pneumoniae* by MALDI-TOF MS; one sample contained *P. aeruginosa*, and the second one contained *S. marcescens and P. aeruginosa* according to MALDI-TOF*.* As our primers have been extensively tested on non–*K. pneumoniae* isolates (including 23 *Pseudomonas spp*. strains), this is likely to be due to the high sensitivity of our LAMP assay, with LAMP having previously been described as suitable for the early diagnosis of infectious diseases (Garg et al., 2021). The *bla*_KPC_ LAMP assay showed a 100% concordance with PCR and antibiotics susceptibility, even in clinical samples. Furthermore, the assays described here demonstrated a limit of detection 100 times lower than the routine PCR method used in Ruijin Hospital, and LAMP results were obtained in a much shorter time frame: 1 h from sample processing to results versus 2 h 30 min for PCR detection and 24–48 h for culture-based methods. Several LAMP assays have been developed for the detection of *K. pneumoniae* (Kang et al., 2012; Dong et al., 2015; Barnes et al., 2018; Li et al., 2019; Tian et al., 2019; Vergara et al., 2020) using different target genes, including KPN_04473 *fimD* (Kang et al., 2012; Barnes et al., 2018; Vergara et al., 2020), *ure1* (Li et al., 2019), *celB* (Tian et al., 2019), and *rscA* (Dong et al., 2015). These different target genes were chosen following bibliographic reviews or sequences alignment and online BLAST searches. The target gene (*yhaI*) described in this work was chosen following a comprehensive comparative genomics study that demonstrated the specificity of *yhaI* to *K. pneumoniae* and its presence in >7,000 *Kp* genomes distributed globally. The designed *yhaI* LAMP assay was validated with high sensitivity and specificity on 5 *Kp* type strains, 167 *Kp* clinical isolates, 32 non-*Kp* type strains, and 115 non-*Kp* clinical isolates from both the United Kingdom and China. Previous studies have also shown the efficiency (high sensitivity, specificity, and concordance with PCR) of LAMP assays for the detection of the carbapenemase families: *bla*_KPC_, *bla*_NDM_, *bla*_OXA-48-like_, *bla*_VIM_, and *bla*_*IMP*_ (Qi et al., 2012; Solanki et al., 2013; Cheng et al., 2014; Nakano et al., 2015; Kim et al., 2016; Srisrattakarn et al., 2017; Funashima et al., 2018; Feng et al., 2021). However, most of these studies used different detection methods, such as fluorimetry, turbidity, and gel electrophoresis, which require specific equipment or repeated lid opening operations (potential cause of cross-contamination) (Qi et al., 2012; Solanki et al., 2013; Cheng et al., 2014; Nakano et al., 2015; Kim et al., 2016; Funashima et al., 2018). LAMP assays, developed using hydroxynaphthol blue dye for colorimetric detection, showed high sensitivity and specificity but used an in-house LAMP Mastermix for which operational factors during reagents preparation may increase the unreliability of the results (Srisrattakarn et al., 2017). A recent study used the same the commercial colorimetric LAMP Mastermix than in our study (Feng et al., 2021) to detect carbapenemase-producing genes; however, their assays were not validated on clinical samples.

Our assays are also comparable to the commercialized CE-IVD LAMP Eazyplex SuperBug kits (complete A, complete B, and CRE; Amplex-Diagnostics GmbH, Germany), designed to detect carbapenemase-producing genes and selected ESBL genes in up to 30 min using the portable Genie II device (Vergara et al., 2014; Findlay et al., 2015; Hinic et al., 2015; García-Fernández et al., 2016; Fiori et al., 2019; Moreno-Morales et al., 2020; Sekowska et al., 2020; Vergara et al., 2020; Holma et al., 2021). Sensitivity and specificity of the Eazyplex SuperBug kits, assessed on clinical isolates of Enterobacteriaceae (Vergara et al., 2014; Findlay et al., 2015), were greater than 95%. These kits were also tested on bronchoalveolar lavage fluid samples spiked with Enterobacteriaceae and carbapenem-resistant *Acinetobacter* spp. (Moreno-Morales et al., 2020; Vergara et al., 2020), clinical urine samples (Hinic et al., 2015), and rectal swab samples (Holma et al., 2021), with 80%–100% sensitivity, a specificity greater than 95%, and a limit of detection between 10^2^ and 10^3^ CFUs/ml (Hinic et al., 2015; Moreno-Morales et al., 2020; Vergara et al., 2020; Holma et al., 2021). Other CE-IVD marked portable molecular diagnostics for carbapenemases, which are cartridge-based PCR assays and require minimal sample preparation, such as Xpert Carba-R (Tenover et al., 2013; Anandan et al., 2015; Findlay et al., 2015; Vergara et al., 2020) and Novodiag CarbaR + combining real-time PCR and microarray technologies (Girlich et al., 2019; Holma et al., 2021), have been recently commercialized. Both these diagnostic methods detect and differentiate the most prevalent carbapenemases gene families in less than 2 h, from samples to results, and showed high sensitivity and specificity in clinical samples with a limit of detection of 10^2^ CFUs/ml (Tenover et al., 2013; Anandan et al., 2015; Findlay et al., 2015; Girlich et al., 2019; Vergara et al., 2020; Holma et al., 2021). These rapid molecular diagnostics are all highly sensitive and specific but require the use of specialized costly devices and reagents. Our designed assays are as sensitive and specific, while being a lot less expensive as it can be performed using only a dry bath heating block. It could be used at point of care as it only requires a heating block and could easily be adapted into different diagnostics platforms, with a potential for mobile connectivity (Barnes et al., 2018; Rohaim et al., 2020). The reagents also have the potential to be lyophilized for use in resource limited areas where refrigeration is impractical.

The emergence of carbapenem-resistant *K*. *pneumoniae* (Wong M. H. Y. et al., 2018; Zhou et al., 2020) has created a new challenge in rapid detecting and combating this already dangerous pathogen. Early diagnosis and intervention could be beneficial in a number of clinical contexts. In urinary tract infection cases (among the most common types of infection) (Flores-Mireles et al., 2015) and pneumonia cases (Beutz and Abraham, 2005); early diagnosis and targeted treatment could help to avoid progression into more severe and potentially fatal conditions such as sepsis (Beutz and Abraham, 2005; Simerville et al., 2005; Seymour et al., 2017). Sepsis cases are difficult to diagnose and constitute a medical emergency, as they progress rapidly, and delay in effective antimicrobial treatment has been associated with an increase in in-hospital mortality (Gaieski et al., 2010; Ferrer et al., 2014; Garnacho-Montero et al., 2014; Martin-Loeches et al., 2015). To improve the survival rates of patients with sepsis, treatment has to be administered within the first few hours (0–6 h) of diagnosis (Gaieski et al., 2010; Ferrer et al., 2014; Garnacho-Montero et al., 2014; Martin-Loeches et al., 2015). Antibiotic therapy usually starts with the administration of broad-spectrum antibiotics, until the source of infection has been identified, at which point a more targeted agent can be used. Broad-spectrum agents are not always very effective, driving emergence and spread of AMR, which is currently one of the biggest global public health threats facing humanity. It is estimated that by 2050, 10 million lives a year and a cumulative US $100 trillion of economic output are at risk from AMR (Brogan and Mossialos, 2016; O’Neill, 2016; Piddock, 2016). Antibiotic effectiveness needs to be preserved as a further increase in AMR would lead to significant risks linked to key medical procedures (such as invasive surgeries, chemotherapy, intensive care, etc.) and infectious diseases management. Rapid identification of the causative pathogen and its AMR, using the assays detailed in this study, would aid in the selection of appropriate treatment, thus reducing the unnecessary use of antibiotics and thereby slowing down dissemination of AMR (Brogan and Mossialos, 2016; O’Neill, 2016; Piddock, 2016), as well as improving the prognosis of septic patients. Our current LAMP assay has been developed for the detection of *K. pneumoniae*. Additional investigations are being pursued to design similar LAMP tests for *E. coli*, a particular concern for AMR (Alvarez-Uria et al., 2018) and a microorganism commonly responsible for sepsis (Restrepo-Alvarez et al., 2019).

## Data Availability

The original contributions presented in the study are included in the article/Supplementary Material, further inquiries can be directed to the corresponding authors.
