# Supplementary material for: Development of Loop-Mediated Isothermal Amplification Rapid Diagnostic Assays for the Detection of Klebsiella pneumoniae and Carbapenemase Genes in Clinical Samples
Source: Front Mol Biosci. 2022 Feb 9;8:794961. doi: 10.3389/fmolb.2021.794961 (PMC8864245; doi:10.3389/fmolb.2021.794961)
Supplement: Supplementary file 2 [file Table7.docx]

**Supplementary Table 6: Summary of LAMP and PCR results for the detection of *yhaI* and *bla*_KPC_ genes on clinical sputum samples, in comparison to gold standard methods.** A total of 40 clinical sputum samples were tested by MALDI-TOF, for bacterial identification. After culture, carbapenems susceptibility was tested on the *K. pneumoniae* strains isolated from the sputum samples. The sputum samples were also tested, after DNA extraction, by LAMP and PCR for the presence of the *yhaI* gene (*K. pneumoniae* specific) and *bla*_KPC_ genes (most frequent carbapenemase). For the LAMP and PCR tests, the results were positive (+) or negative (-), and for the carbapenems susceptibly tests, the results were resistant (R), susceptible (S), and not tested (NT).

| **Sample** | **MALDI-TOF MS results** | ***yhaI* LAMP** | ***yhaI* PCR** | **Carbapenems susceptibly** | ***bla*_KPC_ LAMP** | ***bla*_KPC_ PCR** |
| --- | --- | --- | --- | --- | --- | --- |
| 1 | None | - | - | NT | NT | NT |
| 2 | *Staphylococcus aureus*  *Pseudomonas aeruginosa* | - | - | NT | NT | NT |
| 3 | None | - | - | NT | NT | NT |
| 4 | *Stenotrophomonas maltophilia* | - | - | NT | NT | NT |
| 5 | *Pseudomonas aeruginosa* | + | + | NT | NT | NT |
| 6 | *Pseudomonas aeruginosa* | - | - | NT | NT | NT |
| 7 | *Acinetobacter pittii* | - | - | NT | NT | NT |
| 8 | *Enterobacter cloacae* | - | - | NT | NT | NT |
| 9 | *Acinetobacter baumannii* | - | - | NT | NT | NT |
| 10 | *Proteus mirabilis*  *Pseudomonas aeruginosa* | - | - | NT | NT | NT |
| 11 | *Klebsiella pneumoniae* | + | + | S | - | - |
| 12 | *Streptococcus viridans*  *Neisseria sicca* | - | - | NT | NT | NT |
| 13 | *Klebsiella pneumoniae* | + | + | R | + | + |
| 14 | *Acinetobacter baumannii* | - | - | NT | NT | NT |
| 15 | None | - | - | NT | NT | NT |
| 16 | *Pseudomonas aeruginosa* | - | - | NT | NT | NT |
| 17 | *Streptococcus viridans*  *Neisseria sicca* | - | - | NT | NT | NT |
| 18 | *Pseudomonas aeruginosa* | - | - | NT | NT | NT |
| 19 | *Pseudomonas aeruginosa* | - | - | NT | NT | NT |
| 20 | *Serratia marcescens*  *Pseudomonas aeruginosa* | + | - | NT | NT | NT |
| 21 | None | - | - | NT | NT | NT |
| 22 | None | - | - | NT | NT | NT |
| 23 | *Proteus mirabilis*  *Pseudomonas aeruginosa* | - | - | NT | NT | NT |
| 24 | *Acinetobacter baumannii* | - | - | NT | NT | NT |
| 25 | *Klebsiella pneumoniae*  *Stenotrophomonas maltophilia* | + | + | R | + | + |
| 26 | *Klebsiella pneumoniae*  *Staphylococcus aureus* | + | + | R | + | + |
| 27 | *Klebsiella pneumoniae*  *Proteus mirabilis* | + | + | R | + | + |
| 28 | *Klebsiella pneumoniae* | + | + | R | - | - |
| 29 | *Klebsiella pneumoniae* | + | + | S | - | - |
| 30 | *Klebsiella pneumoniae* | + | + | S | - | - |
| 31 | *Klebsiella pneumoniae* | + | + | R | + | + |
| 32 | *Klebsiella pneumoniae* | + | + | S | - | - |
| 33 | *Klebsiella pneumoniae*  *Pseudomonas aeruginosa* | + | + | S | - | - |
| 34 | *Klebsiella pneumoniae*  *Staphylococcus aureus* | + | + | S | - | - |
| 35 | *Klebsiella pneumoniae*  *Pseudomonas aeruginosa* | + | + | R | + | + |
| 36 | *Klebsiella pneumoniae*  *Pseudomonas aeruginosa* | + | + | R | + | + |
| 37 | *Klebsiella pneumoniae* | + | + | R | + | + |
| 38 | *Klebsiella pneumoniae* | + | + | R | + | + |
| 39 | *Klebsiella pneumoniae*  *Proteus mirabilis* | + | + | R | + | + |
| 40 | *Klebsiella pneumoniae* | + | + | R | + | + |
