# Supplementary material for: Development of Loop-Mediated Isothermal Amplification Rapid Diagnostic Assays for the Detection of Klebsiella pneumoniae and Carbapenemase Genes in Clinical Samples
Source: Front Mol Biosci. 2022 Feb 9;8:794961. doi: 10.3389/fmolb.2021.794961 (PMC8864245; doi:10.3389/fmolb.2021.794961)
Supplement: Supplementary file 3 [file Table2.docx]

**Supplementary Table 2. Bacterial isolates tested to evaluate the cross reactivity of the LAMP assay.** Clinical: fully characterised clinical isolates from partner hospital; ATCC: American Type Culture Collection; NCTC: National Collection of Type Cultures, Public Health England.

| **Organism** | **Reference** |
| --- | --- |
| *Pseudomonas aeruginosa* | PAO1 |
| *Pseudomonas aeruginosa* (20) | Clinical (Ruijin Hospital) |
| *Pseudomonas aeruginosa* | MPAO1 |
| *Pseudomonas putida* | KT2440 |
| *Enterobacter cloacae* | NCTC 13380 |
| *Enterobacter cloacae* | NCTC 14322 |
| *Salmonella enterica* serotype Typhimurium | ATCC 14028 |
| *Salmonella enterica* serotype Pullorum | NCTC 5776 |
| *Salmonella enterica* serotype Pullorum | NCTC 10705 |
| *Salmonella enterica* serotype Gallinarum | NCTC 13346 |
| *Salmonella enterica* serotype Gallinarum | NCTC 10532 |
| *Salmonella enterica* serotype Typhimurium (2) | Clinical (Leicester Hospital) |
| *Salmonella enterica* serotype Newport | Clinical (Leicester Hospital) |
| *Salmonella enterica* serotype Infantis | Clinical (Leicester Hospital) |
| *Salmonella enterica* serotype Chester | Clinical (Leicester Hospital) |
| *Salmonella enterica* (20) | Clinical (Ruijin Hospital) |
| *Escherichia coli* | NCTC 14321 |
| *Escherichia coli* | ATCC 25922 |
| *Escherichia coli* | NCTC 13441 |
| *Escherichia coli* | DH5α |
| *Escherichia coli* (20) | Clinical (Ruijin Hospital) |
| *Acinetobacter baumanii* | ATCC Ab19606 |
| *Acinetobacter baumanii* (20) | Clinical (Ruijin Hospital) |
| *Acinetobacter iwofii* | NCTC 5866 |
| *Staphylococcus aureus* | NCTC 12981 |
| Methicillin-resistant *Staphylococcus aureus* (MRSA) | NCTC 12493 |
| *Staphylococcus aureus* | N315 |
| *Staphylococcus aureus* (20) | Clinical (Ruijin Hospital) |
| *Staphylococcus aureus* | NCTC 8325 |
| Methicillin-resistant *Staphylococcus aureus* (MRSA) | BAA-1680/USA300 |
| *Yersinia enterolitica* | NCTC 12982 |
| *Streptococcus pyogenes* | NCTC 12696 |
| *Klebsiella aerogenes* (5) | Clinical (Ruijin Hospital) |
| *Klebsiella oxytoca* (5) | Clinical (Ruijin Hospital) |
| *Klebsiella oxytoca* (9) | Hospital environment (sink drain traps of Lancashire NHS hospital) |
