# Supplementary material for: Development of Loop-Mediated Isothermal Amplification Rapid Diagnostic Assays for the Detection of Klebsiella pneumoniae and Carbapenemase Genes in Clinical Samples
Source: Front Mol Biosci. 2022 Feb 9;8:794961. doi: 10.3389/fmolb.2021.794961 (PMC8864245; doi:10.3389/fmolb.2021.794961)
Supplement: Supplementary file 6 [file Table6.docx]

**Supplementary Table 6. Genes sequences selected to create a custom ABRicate database**, used for *K. pneumoniae* genomes screening.

| ***Gene name*** | **Gene sequence** |
| --- | --- |
| ***DgoK1_1*** | **>klebsiella~~~Kp00840_01120~~~DgoK1_1**  ATGAACGAGTATATTGCCGTCGATTGGGGCTCCACCCAGCTGCGGGCATGGCGGATGCGT  GATGGCGAATGCATCGACAAGCTGAAACTGCCCTGCGGCGTCACGCGTCTGAACGGGCAG  CGCGCCGAAGCGGTATTTCAGCAGCAGTTGGCGCCGTGGCGCGGCGACCCCGCGCTGCCG  GTGGTGATGGCGGGCATGATCGGCAGCGATGCCGGTTGGCAACCGGTGCCTTATCTGGCC  TGCCCGCTGGCGCTGGAGGCACTCAACGGTCAGTTATATGAGGTGGCGGAAAAAGTGTGG  ATCGTGCCGGGGCTGAAAGTGGCGCAGGCTGCAGATTACGACGTAATGCGCGGTGAAGAA  ACGCAGCTGCTGGGCGCCTGGCAACTGATGCCCGCGGAGTGTTACGTGATGCCCGGCACC  CACTGCAAATGGGTGCAGGTGCAAAACGGCGTGGTGCGCCAGTTCGCCACGGCGATGACC  GGCGAACTGCATCACCTGCTGCTTAACCATTCGCTGCTCGGCCAGCAACTGCCTGCGCAG  TTCCCGGATGAAGCCTCTTTCGCCCTCGGTATAGAAAAAGGGTTAAATCAGCCGGCGTTA  TTGTCCGGGCTTTTTAGCGCCCGCGCCGCGCGGGTGTTAGGCTCGCTGGCGGCGACTTCG  GTGAGCGATTATCTCTCCGGGCTGTTGATTGGCGCGGAGGTGGCGACATTCAGCGAACGC  TATCGCGCCAGCCGCGTGGTGCTGGTTGGTGAGCACTCGCTCAACGCCCGTTATCAGCAG  GCGATGGCGGCGCGTGGGTTAGCCGTTTCCTGTTGCTCCGGCGAGGCGGCGTTTCTTTCG  GGTATAGCGAGGATGATTGATGGACAAGATTAA |
| ***creD*** | **>klebsiella~~~Kp00840_01750~~~creD**  ATGTTGAAATCACCGTTATTCTGGAAAATCACCACGTTAATAGGCTGTATTGTGCTGCTA  TCTTTGCCGTTAATGATGGTCAGGGAGCTTATTAATGAGCGAGCGGATTATCGTAACGAA  GTGGTGGACGCCATTGAGCAAAGCACCAGCGGCTCGCAAAAACTCGCCGGACCGCTGATC  GCTATTCCGATAACCGAGACCCTGACCCGCATGGAGAACCAAAAAGAGGTCAACTATCAG  CGGAGCTGGGTGTATTACTGGCTGCCGGAGTCGCTGGCGGTCGCCGGCAAGCAGACCGTG  GAGTCGAGGAGGGTGGGTATCTACAGCGGCCAGGTCTGGCATAACGTCTTGCAGATTAAG  GCCTCGTTTGATCCGCTGCGTCTGGCGGCGCTGAGAAAAACGAATATCGTCTTAGGCCAG  CCGCGGCTGGTGGTGAGCGTCGGCGATGCCCGCGGCATTGGCGCCATCCATGCCCCGGAG  GTCAACGGTAACGTATTAAGCGTGGAGCCGGGGCTGGGGATAAGCGGCGATGGCGCAGGT  ATTCATATGCCAATGCCTGCGCTGGCGGAGGACAACAAGCCGCTTGAGATTGCCTTTTCA  CTCGATCTGAACGGGACGGGTGAGTTTTCACTGGTGCCGCTCGGGCGTAACAGCGAGCTG  CAGCTGACCAGTAACTGGCCGCATCCCGGTTTTCTCGGCAGCTTCCTGCCAACGCAACGG  GAAGTGAGTGCTGCCGGCTATCGCGCCCACTGGCAAAGCAGCTGGTTCGCCAACGATATG  GGGAGCTATTTCAAGGATGATATGGAGATCCCGTGGTCGCGACTGCCGGCGTTCAGTGCC  GATGTGATGTCCCTTGCCGACCAGTACCAGCTAACCGATCGGGCGACAAAATATGCCATC  CTGTTAATCGGGCTGACCTTTATGGCTTTTTTCGTGTTCGAGAGCCTGACCCGTCGCCCC  CTGCATCCGATGCAATATCTGCTGGTGGGCTTATCGCTGGTGCTGTTTTACCTGGTGCTG  CTGGCGCTCTCAGAGCACATTGGTTTTACCGCCGCCTGGCTGGCGGCCAGCCTGTCTGGC  GCGGTGATGAATGGGATCTACCTTCAGGCGGTGCTCAGAGGATGGCGTAACAGCCTGCTG  TTTGTCGCCGCGCTACTGCTGCTGGATGGCGTGATGTGGTTTCTGTTGCATTCTGAGGAT  AGCGCGCTGCTGCTGGGTACCGGGGTGCTGGCGCTGGCGCTGAGCGTTCTGATGTTTTTG  ACCCGTCGCGTGGACTGGTATGCGTTATCGCTGCCGAAAGGCACAGTGCCTCCGACACCG  GCTGCCGATGACGATAAGCTGCGTCTCTGGAAAGAGTGA |
| ***yhaI*** | **>klebsiella~~~Kp00840_02010~~~yhaI**  ATGACCTATGGACAGGCGTATTTGAGCGGCTGGAAAGAGACCTTTAACTTTAGCGGACGC  GCCAGTCGACGGCAGTTCTGGACGTTTTTTCTCACCAATGTATTGATCGCCACCGCGCCG  CTGGCGGCCTGGTGTCTGGCAACCAGCGTTAACCCTCAATACGGCATCCTCAGTTTTGTT  GTCATTCCCTTTGCCGCGCTCTGGCTGCTGTTGATGGCGATCCCCCTGCTGGCTGTAGGC  TGCCGGCGGATGCATGATATCGGCCGCTCCGGGATCTGGTTTGTGTTGGGGGTTATCATT  CCGTGGTTTGCAATTATTTCGCTGGCTCTGTGCTGCCTGCGCTCAGCGCCGGCACCGTCT  CGCTGA |
| ***YebZ*** | **>klebsiella~~~Kp00844_07280~~~YebZ**  ATGCTGACCGGGCTCTATATTACCCTGCGCTTTGGGCACTTTATCTCCCTGATGCTGGCT  TTTGGCTGCGTGCTGTATGGCGCCTGGTGGGCGCCGGTACCGCTGAGACGCGTGCTGATG  CTGCGCTTTTATCCACTACTGCGCCCGCTGCTGCTGATCGGCGCGATCTCTACGCTGGCG  CTGTATCTCCTGCAGGGAGGAATAATGGGCGAGGGCTGGACGGATGTCTGGCGACCCGCC  GTCTGGCAGGCGGTGGCAGGGACCCGCTTTGGTGGAGTGTGGATCTGGCAGATCCTACTG  ACGTGGATCGCACTTGCGGTAGTGTGGATCCGTCCCCGTCACGGCGCCCGCCAGCTGGTG  GCCCTGCTGGCGGCACAGCTGCTGCTTTCCGCCGGGGTAGGGCACGCCGCTATGCACGAT  GGTCTCACCGGCGTGCTGCAGCGGACAAACCACGCTGTGCATCTCTTCTGCGTCGCCAGC  TGGTTTGGCGGTCTGTTGCCCTTTATCTATTGCCTGCGCCTGGCGCAGGGGCGCTGGCGG  CCGGCCGCGGTCTATACCATGGCGCGCTTTTCACGTTACGGGCATCTGGCCGTCGCCGGC  ACGATTGCCAGCGGAGCGCTCAATGCGCTGTTGATCCAGGGAGGATTGATTGGCGCATCG  CCGTGGGGACGCATGTTGTTGATCAAATGTGCGCTGGTCGCCGGGATGGTGGTAATTGCG  TTAGTGAACAGGTATGTTCTGGTACCGCGCATGTCGGCAAGCGGTTCGCGGGCGGAAAGC  CTGATCCTGCGAACCACGCAGGCTGAAATAGGGCTGGGCGCGCTGGCGCTGCTGGCCGTC  AGTCTGTTTGCCACCTGGGAACCTTATTGA |
| ***pphA_2*** | **>klebsiella~~~Kp00844_07320~~~pphA_2**  ATGTATCAACGTATTAACGGCAGCGACTGGCGTAATATCTGGCTGATGGGCGATCTGCAT  GGCTGCTTTGCGCTGCTGATGAATCGCCTGCGTCAGCTGCGTTTTGATCCCTGGGCCGAT  CTGCTGATCTCGGTGGGCGATCTGATCGATCGCGGGCCGCAGAGCGCCGATTGTCTTGGC  CTGTTGCGCTGTCGCTGGTTCAGGGCCGTTCGGGGGAATCATGAGCAGATGGCGCTGGAG  GCGCTGGAGAGCGGAGATATGCGGCTCTGGCAGATGAACGATGGCGACTGGTACGTCAAG  GGCGATGCGCGGCAGCGGGCCGACGTCGATCGGCTGCTGGCGCACTGTCGGCGGCTACCG  TTGATTATTGAAGTGCAATGCGAGAAAGCGCGGCATGTGATCGCCCACGCCGATTACCCG  GCGCCGGTCTATCGCTGGCAGCAGCCGGTGGATCCCCAACGGGTACTATGGAGCCGCCAT  CGTCTGAGTGAACACCTGGCGGGGCGCCATGGCGCTATTGGCGGCGCAGACCATTTCTGG  TTTGGCCATACGCCGCTTCAGGCGCGTTATGACCACGATAATCAACACTATATTGATACG  GGCGCTGTGTTTGGTGGGACGCTGACGCTGGTGGCGCTGCAGTCGGCAGGCTAA |
| ***DsdC*** | **>klebsiella~~~Kp00844_23880~~~DsdC**  GTGACCGCCCGCCACCTGAGCTTTACCCTTGCCGCGCATGAAATGAACCTTACCCAGGGA  GCGGTCAGCCATCGGATCCGCAGGCTGGAGATGCACATCGGGTTTCGCCTGTTTATCCGC  ATGACGCGTAAACTGGCGCTGACAGAGGAAGGTAAGCGGCTGCTGGCGACGTTAAGCCAT  TCGCTGCGGGCGATTAACGATGAAATTGAGGATATTCGCGATCAGGATCTGCGCGGCACG  CTGCATATTGGTATTGCGCCGACGCTGGCCCATCTGTGGCTAATGCCGCGTCTGCCGCGC  TTTCAGACGCAATGGCCGGGACTGAATCTGCAGTTTCGCGTGCGCGCCGGGGTGATGGAT  TTTAACGAGGAGCGGGTGGATCTGGCAATTTACTACGGCGCCACGCGCTATCCCGATCTT  TATCAACAGCGGCTGATGGCGGAAAGTTTGCTCCCGGTCTGCTCCCCGCGTTATCGCCAG  CAGTATCGTCCGCTGAGCGGCGGCGACCCGGCGGCGCTCGTCTGGATCCATGCGTGCGAA  TCCACCGACGTACAGGATCAATTTGCGGAATGGCGCCTGTGGTGCCAGCACAGCGGACAG  GCGTTACCTTTTGACGGCCGGTATTACGCGGTGAATAACCACTCGCTGGCCATCGAGATG  GCGCTCAATGGACTCGGCGTGGTGATGGGGCGTAAAACGCTGATCCAGCCGCTGCTCGAC  GCCGGCAGGCTGGTGGCCTTGTCTGAAAATGAAGCGCCCTCGCCCTTTGGCTATGACCTG  ATTTGTCCGCAGGAGAACCGTTCCCGCCCGCGCTTTCGCGCCTTCAGCGAGTGGCTGGCG  GCGGAATGCGCCTAA |
| ***epsL*** | **>klebsiella~~~Kp00844_46720~~~epsL**  ATGCTGATCGTGCGCCTCGCCGCCGCGCAGGCGCCGCTGCACTGGCAACTCTTCGCCCCC  GGCGAGCCGCACCACGAGGCCAGCGGCCGGTGGCCGACTGACGACGCCAGCCCTTTCCCG  GCGCTGGCTGAGCAGTACCCGGCCTGGGTGCTGATCCCGGCCAGCGACTGCGCGTTTCAC  TCGCTCAACCTACCGGCGGGCCTGCGTAAACCGCCGCTGCAGGTGGCCCCGTTTCTGCTG  GAAGAGCAGCTGGCAGACGATGTGGAAGCCACCCATTTTGCTCTGCTCCACCGCCAGCAG  GCCCAGTGCGAGATCGTCGCCGTACAGCGGCAAAAAATGCGCGACTGGCTGGCGCGCTGC  GAATCGCTCTCCTTACAGCCCCTGGCGTTGACGCCCGACGTGCTGGCCCTGCCCTGGCAA  CCGCCGGCGTGGAGCGCGGTGCAGGTGGACGAGCAGTGGCTGATCCGCCACCAGCCGTGG  GGCGGCATGGCGGCGGAAAACGTCTGGCTGACGGAGCTGCTGCAGAGCGAGGCGGAAGAG  CACGTCATCGACAGCTATTCGCCGCCGCCCGCGGCGCCGGGCGTCTGGCGGGAGCAGCCT  GCGCAGACATTGCTGACCCTTGCCGCACGCCATCCGGCAGCGCAGAAACTCAGTCTGCTG  CAGGGGGAGTTCGCCGTCCGGCGGCGGTCTGCGCAGGCGAGCTGGCGTCCGGCGCGGTAT  GCCGCGCTGGCTCTGGCGTTACTGGCAGGGGCAAACAGCGTGCTGGACCATCGCGATCTG  GCGCGTCAGGCCGAGGCGGCGCAACAGGCCAGCCGGGCCTTCTATCACCGCTGGTTTCCG  ACGGAGAAAAAGGTGATTAACCCACGACTGCAGATGCAGCAGCACCTGCAAACCCTCACC  CGTCAGGCGCAGCATGCCCCCCTCGTCGACCGCCTCAGCGCCTTGCAAAACATCCTCAGC  GAAACGCCGGGGATCCGTCTGCGAGTGCTGAGCTGGGATGCCGCGGGCAATCGTCTGCAG  CTGGATATCGCCGCGGTCACTTCCCGGGCGCTGGAGCAGTTTACCCAGCGGGCGCAGCCG  CGGTTTCGCGTCCGGCCGGGCGACATGATCACGAAACCGGATGGCATTGAGGGACAACTG  ACGCTGGAGGAAAACGATGGCTAA |
| ***xcpW*** | **>klebsiella~~~Kp00844_46740~~~xcpW**  ATGATAACGAAAATACGCGGTTTCACTCTGATCGAAACGCTGCTGGCGCTGGCTATCCTC  GCCGTCCTCAGCGCCGCCGCCGTGATGGTGCTGCAGAACGTCATCCGCGCCGATGGCCTG  ACCCGCGAGAAAAGCCAGCAGATCGCGGCCCTCCAGCGCGCCTTTCGCCAGATAGCCGAC  GATGTCACCCACATCATTCCCCGTCGCGCCAGAAACAGCGACACGTTTTTCTTCGCCGGA  CGTTTCCAGTTGCAGAGCGACGACTGGGGACTGGCCTTCAGCCGCAGCGGCTGGCCAAAC  CCGCTGGGGATCCTCCCGCGCTCGGAGATCCAGAACGTCAGCTACCGTCTGCGCCAGCAG  CAGTTTGAACGTCTGAGCTTCGATCAACAGGATCCGCTGACCGGCAGCCAGCCGACGGTC  CGGGTGCTGCTGCGCGAGGTCACAGCGTTTCGCCTGCGGTTTTACGCCGACGGGCGCTGG  CAGGAAACCTGGGACCGCCCGCAGAGGCTGCCGCAGGGGCTGGAGATCACCCTGACGCTG  GCGAACAGCGGGGAGATAACCCGCCTGTTTTTACTCACCCCGGGAGGCGGCCAGTGA |
| ***outB*** | **>klebsiella~~~Kp00844_46830~~~outB**  GTGATTGTTCGTGATGACAGTGAAAGAGGCATCCCGCCGCTGGTGACGCATCAGCCGCCG  TCCTCGATGGATGATGCGCCGGTGATCCGCGGACGGATGGTGCAGATCCCCGGCTGGATT  ATCCCGCTGTACGCCGGATTATTTATCGCGCTCGGCTGGTTTGGCGGCGAGCAGTGGCGT  CACCCGGCACCGCCGCAAACGCTGCCCTTGCCGGTGGCGCATGCGGCGCTTCTGCCGTTA  ACCGCTGCCGGAGAGGCCGCGCCAAAGACGCCGCCGCCGGCGGCAAAAGCCAGCGCGCCC  GCGGCGCCGGAAGTGGACAGCGATACGCTGCCGCCTCTACGCTACAGCGCCCATGTCTAT  GCCTCGCTACCGGAAAAGCGCAGCATTGTGTTAAACGGCAAAGCGTGGACGGAGGGCGAT  TCACCCCTGCCCAATCTGGTGGTGGAGCAGATCCAGCAGGATGTGACGATCTTCAGCTTC  AACGGCACGACCTTTACCCTCGCGGCGCTGGAGGACTGGCCGGGCGGAAAAATTGACGAG  GAGCCGAAAGAGGAATAA |
